# Exploring How White-Faced Sakis Control Digital Visual Enrichment Systems

**DOI:** 10.3390/ani11020557

**Published:** 2021-02-20

**Authors:** Ilyena Hirskyj-Douglas, Vilma Kankaanpää

**Affiliations:** Department of Computer Science, Aalto University, 02150 Espoo, Finland; vilma.kankaanpaa@aalto.fi

**Keywords:** white-faced saki, animal-computer interaction, animal technology, visual enrichment

## Abstract

**Simple Summary:**

Many zoo-housed primates use visual computer systems for enrichment but little is known about how monkeys would choose to control these devices. Here we investigate what visual enrichment white-faced saki monkeys would trigger and what effect these videos have on their behaviour. To study this, we built an interactive screen device that would trigger visual stimuli and track the sakis’ interactions when using the system. Over several weeks, we found that the sakis would trigger underwater and worm videos significantly more than animal, abstract art and forest videos, and the control condition of no-stimuli. Further, the sakis triggered the animal video significantly less often over all other conditions. Yet, viewing their interactions over time, the sakis’ usage of the device followed a bell curve, suggesting novelty and habituation factors. As such, it is unknown if the stumli or devices novelty and habituation caused the changes in the sakis interactions over time. These results also indicated that the different visual stimuli conditions significantly reduced the sakis’ scratching behaviour with the visual stimuli conditions compared to the control condition. Further, the usage of visual stimuli did not increase the sakis’ looking at and sitting in front of the screen behaviours. These results highlight problems in defining interactivity and screen usage with monkeys and screens from looking behaviours and proximity alone.

**Abstract:**

Computer-enabled screen systems containing visual elements have long been employed with captive primates for assessing preference, reactions and for husbandry reasons. These screen systems typically play visual enrichment to primates without them choosing to trigger the system and without their consent. Yet, what videos primates, especially monkeys, would prefer to watch of their own volition and how to design computers and methods that allow choice is an open question. In this study, we designed and tested, over several weeks, an enrichment system that facilitates white-faced saki monkeys to trigger different visual stimuli in their regular zoo habitat while automatically logging and recording their interaction. By analysing this data, we show that the sakis triggered underwater and worm videos over the forest, abstract art, and animal videos, and a control condition of no-stimuli. We also note that the sakis used the device significantly less when playing animal videos compared to other conditions. Yet, plotting the data over time revealed an engagement bell curve suggesting confounding factors of novelty and habituation. As such, it is unknown if the stimuli or device usage curve caused the changes in the sakis interactions over time. Looking at the sakis’ behaviours and working with zoo personnel, we noted that the stimuli conditions resulted in significantly decreasing the sakis’ scratching behaviour. For the research community, this study builds on methods that allow animals to control computers in a zoo environment highlighting problems in quantifying animal interactions with computer devices.

## 1. Introduction

Many modern zoos incorporate technology into their facilities through computer enrichment and monitoring systems [1,2,3,4,5,6]. These computers are used to inform and extend humans’ understanding of animals’ behaviour and capabilities and to provide enrichment for the animals [7]. Enrichment systems used by animals aim to improve or enhance the animals’ quality of life (i.e., [2,3,6,8,9,10,11]), support species-appropriate behaviours [12] and ensure the animals’ natural behavioural competences [13]. Computer-enabled enrichment systems seek to do this by giving animals control over their activities and environment [1,14]. Most of these computer-enabled enrichment systems are for primates, for example, music systems for apes [6,15] and monkeys [7], motion tracking to allow apes to play with projections [3,11,16] and a feeding puzzle for gorillas [2]. Moreover, beyond these examples, most of these computer-enabled enrichment systems have been used with great apes and involve tasks on screens [1,16,17,18], that encourage comparable physical and cognitive processes and behaviours in the captive species to their wild counterparts in natural habitats [19].

With the focus on screen enrichment systems for primates, these computer devices commonly consist of auditory and visual enrichment through tablets or bespoke screens made for the animal user [19]. These screens provide videos and auditory stimuli as well as cognitive challenges [16,18,19,20,21,22,23], are used for husbandry purposes and for assessing a primate’s food-preference [24], mood, personality and social skills [17].

To understand primates’ experience and usage of screen enrichment, considerable effort has been put into investigating how these animals experience screens and into comparing screens to visual and real-life mediums. In pictures, Capuchin monkeys have been shown to be able to distinguish their counterpart monkeys’ faces [25], preferring red-featured images [26]. However, it has also been hypothesised that inexperienced monkeys confuse realistic pictures with reference objects [27], leading to the belief that referential usage in pictures is found mostly in ape species [28].

Applying these same questions on experience, usability and cognition to computer screens with apes for enrichment, scientists have found that orangutans and chimpanzees prefer auditory interactions and bright colours [20]. Additionally, playing videos to orangutans in their enclosure encouraged social behaviours, increased their activity and reduced their cortisol levels [23]. Chimpanzees’ vision has been demonstrated to be comparable to humans’ vision with screens, and they have been demonstrated to be able to learn from screens, too [29]. For monkeys in particular, researchers have found that macaques interact with screens 40% of the time [17]. Macaques’ also prefer contrafreeloading with screens—undertaking tasks on screens for food rather than being provided food for no effort [17]. Moreover, macaques’ visual behaviours correlate to humans when watching videos on screens by both scene content and context [30]. Screens have also been used with capuchin monkeys and rhesus macaques for cognitive tasks to test choice [31] and ability to learn from videos [32]. Notably, rhesus macaques have been shown to watch videos on screens, with habituation occurring more with males but female monkeys showing more interest, indicating sex differences [33]. Macaques also, after being trained to use screens for choice, will prefer social stimuli over nature documentaries [34]. Mandrill monkeys’ visual perception has also been tested with screens, where research found that they were attracted to biologically relevant colours and shapes [35]. Building on this, researchers have speculated that macaque are motivated by biologically-relevant social stimuli such as the contents expressions and faces, and the bodies and their movements [30] and other psychological factors [34].

Many of these screen enrichment systems for primates are used to provide the animal an element of choice. For zoo-housed animals especially, allowing the animals to have control over their environment through choice (such as light, heat and visual stimulus) has been demonstrated to induce positive behavioural welfare indices, improving the animals’ overall living standards [36]. Still, while there has been much work conducted on choice for animals since the 1980s [37], few computer-enabled enrichment systems give the animals direct control over when and what the system does. In the context of screens and primates, most computer-enabled systems automatically play visual content to primates within their enclosure without their consent [21,23], often motivating their usage with food rewards [18,34] in artificial settings [17]. Besides, most of these enrichment systems for zoos focus on great ape family i.e., [18,19,20,21].

Noting this, Ogura [38] made a video system for Japanese macaques that played different YouTube videos when they approached the screen. They found that the video stimulus reduced abnormal behaviours, had little evidence of habituation and that the monkeys’ watching behaviours differed over the content. These results demonstrate that visual stimulus could be useful for influencing behaviour and improving the welfare for zoo-housed monkeys. However, beyond this study, little is known about the long-term effects of visual enrichment and what monkeys’ usage would look like if the monkeys themselves, un-coerced, controlled the screen device when they wanted to. It remains an open challenge to create methods to quantify a monkey’s free-formed choice with screens in an animal-friendly manner and to develop cost-effective systems to facilitate and automatically capture their interaction and behaviour.

Addressing these gaps in screen systems and choice for zoo-housed monkeys, in this paper, we present a digital visual enrichment system created for and controlled by white-faced saki (*Pithecia pithecia*), hereafter referred to as sakis. We use sakis as an instance because little is known prior about this species and monkeys, and because of ease of access. To allow sakis to control and consent to the playing of visual content autonomously through their everyday behaviour, we designed and built a system that uses body tracking to record and log the animals’ interactions with visual stimuli systematically. In a longitudinal study, we use this visual enrichment system with sakis over several weeks, to explore the sakis’ free-formed usage and behaviours in response to the different stimuli. Through measuring their behaviour we then reflect, with the zoo’s behavioural experts and keepers, upon the sakis’ visual preference and the behavioural effects of controlling a screen.

This study aims to add and form the groundwork for animal enrichment systems and methods for investigating and facilitating monkeys having choice and control over these systems. The developed system in this study is shown to be low-cost and useful in the long-term for tracking monkeys’ behaviour during interaction with the device to explore what happens when monkeys are given some control over a digital visual device. This paper reflects upon the findings and method itself, asking the following research questions:

**RQ1**  **What visual enrichment do captive white-faced sakis trigger?**
**RQ2**  **What are the behavioural effects of visual enrichment upon captive white-faced sakis?**

## 2. Materials and Methods

All of the experimental procedures and methods described below were ethically approved by the Korkeasaari Zoo and caused no pain or discomfort to the animals following the European Act on the Protection of Animals Used for Scientific or Educational Purposes.

### 2.1. Participants

The participants in the study are seven white-faced sakis living in the Amazonian House in the Korkeasaari Zoo. Sakis are medium-sized primates and in the wild live in extremely remote areas typically being shy and moving fast and silently through the forest. These factors lead to them being a poorly studied species, with no behaviour studies published related to their colour vision or vision with computers. However, sakis are largely thought to have colour vision due to sexual dichromatism [39].

The troop of sakis in the study consisted of seven individuals (M = 9.7 years): three males (aged 21, 5, and 4 years) and four females (aged 22, 11, and two 4-year-olds). All the sakis were born in the Korkeasaari Zoo, except the 21-year-old male that was born in London Zoo coming to the Korkeasaari Zoo in 2003, and remain as a family group since. All the males are sterilised surgically, and the older female has a hormonal implant to prevent breeding. This is to improve their welfare since too many breeding animals in a group can cause problems and it prevents the offspring from breeding with their parents. This captive group of seven fit demographically with the range seen in natural social groups in the wild (2–12 [40]). Due to researching in a working zoo environment, the sample size including all the sakis within the enclosure was decided by the zoo with no control group available.

The sakis’ enclosure consisted of two adjacent spaces that are on display for visitors. Their outside enclosure is 6 m by 3 m and 5 m high. Their inside enclosure consists of an area that is 6 m by 9 m approximately and 6 m high at their peak (where the central tree is) and 4 m at its lowest. The inside enclosure consists of a large central tree, various hanging logs and branches, plants and other enrichment devices. There is also bark on the floor. The outside enclosure is similar but with wire mesh on the ceiling. There is also an empty space between these enclosures, that is not visible to the public. This space is approximately 2 m by 2 m with a height of 3 m located inside the keepers’ premises. The sakis themselves can freely move within and between these spaces. The ambient soundscape of their habitat included vocalisations of other animals living in the Amazonian section of the Korkeasaari Zoo where they resided. This consisted mainly of bird calls (mostly parrots) and sounds of the other small monkeys including those co-habitating in their enclosure. The co-habitating monkeys were pygmy marmoset monkeys and emperor tamarin monkeys (for a short period). A Hoffmann’s two-toed sloth also resided within their habitat. Due to researching in a live zoo environment, occasionally, some sakis were moved from the enclosure for short periods of time resulting in different groupings over the study.

This troop of sakis had previous experience with music interactive device [7] and had been trained to touch coloured sticks and to do other cognitive tasks with touch [39]. They had no previous experience of computerised visual interfaces.

### 2.2. Design Requirements

**Figure 1:**
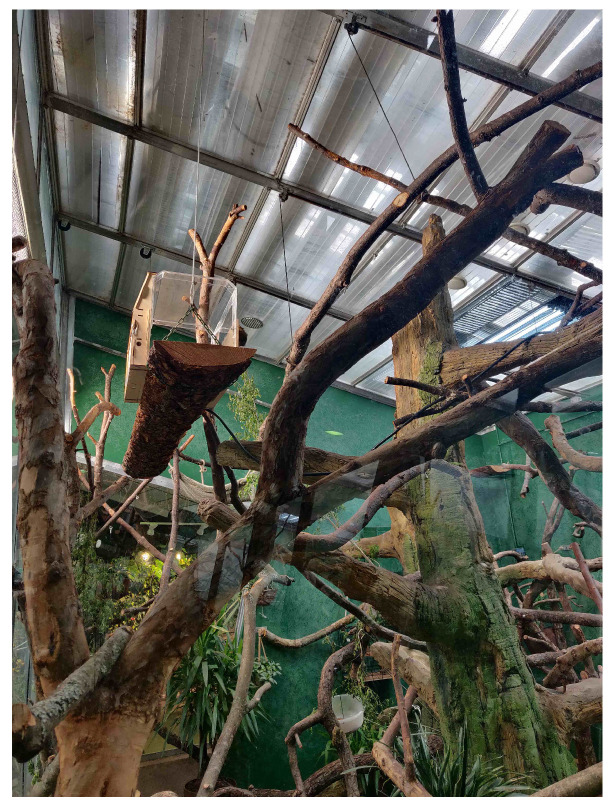
Device inside the sakis’ enclosure at a height of approximately 3.5 m from the ground.

As part of the design of the system, to meet the need and requirements of the sakis, we followed the requirements for zoo-housed sakis formed by Hirskyj-Douglas and Piitulainen [41]. These focus on animal-centric design principals to make technologies that fit the animals’ and zoo’s requirements. Drawing from this, the system’s interaction had to be designed so that the sakis did not have to be trained and the sakis could freely choose how to use the system. This provided the sakis a form of consent and autonomy; the sakis could avoid the system if they chose to do so. Following these guidelines, the device structure itself was built from wood and clear plastic to blend in with the sakis’ current environment (see Figure 1) and to meet their affordances (e.g., no slippery surfaces, large enough for them to fit comfortably inside). The device had no parts that the sakis could eat, was waterproof within the technology compartment, and was designed to be see-through to prevent the sakis feeling enclosed. Inside the sakis’ enclosure the device was placed at a height of approximately 3.5 m due to sakis usually living in trees and not at ground level (Figure 1).

### 2.3. Method

To investigate what visual enrichment the sakis trigger and the effects of this triggering (RQ1 and RQ2), we built an interactive device that could be controlled by the sakis. The device is a tunnel-shaped structure that has a screen, camera and proxemic detectors inside on the wall (see Figure 1). When a saki is detected by the device, the system displays a video (only visual) on the screen inside the structure (otherwise remaining off), and collects the information (video played, time/date, length) of the interaction. In designing this system we considered both how this system could be used and controlled by the monkeys themselves directly, both as a research tool but also as a system prototype. We formed a method building from prior work [7,42] to both allow monkeys to (1) control their interactions with the computer and (2) to help to understand and support the sakis.

The experiment began with a control week (no stimuli) to detect the sakis’ regular movements inside the tunnel for a comparative baseline. After the first week, the system would play the same pre-selected visual stimuli, a video (no sound), whenever the sakis were detected inside the form. If there was not a saki detected inside the structure, the system’s screen would be turned off while the system itself would remain continuously on. After a week, the stimuli would change to another. This method of weekly iterations of playing the same stimulus was repeated in six weekly iterations: no stimuli in week 1, forest stimuli in week 2, underwater stimuli in week 3, worms stimuli in week 4, abstract stimuli week in 5 and animals stimuli in week 6. We used the same visual stimulus for duration of seven days to facilitate the sakis associating the tunnel with the changed stimulus. Furthermore, this long testing period mitigates against changes over time and has been found to be successful and viable at measuring sakis’ behaviour and preferences prior [7].

To confirm the detected interaction, the system recorded it via cameras within and outside of the system, along with the timing, length and date of the interaction. This packaged data was saved locally and was daily uploaded to an online server to allow for remote inspection of the data. The system itself could also be accessed remotely to allow for manual uploads, error handling, and software updates. The system had a feature to report its status hourly to monitor any failures and errors. This resulted in an autonomous system housed within the sakis’ enclosure.

**Figure 2:**
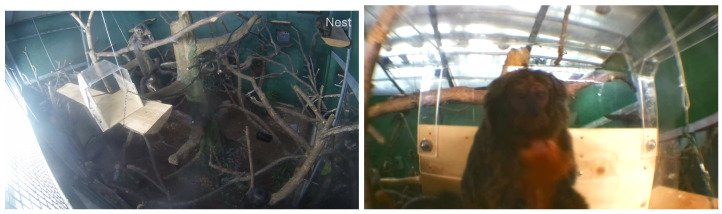
The view from Google Nest and Raspberry Pi cameras.

The camera outside of the system was Google Nest camera owned by the Zoo, having a view over the sakis’ enclosure. This video feed was set to track the monkeys’ movements within the system’s space. This external video was saved and used to verify the system’s video from inside the tunnel, and to see the monkeys’ behaviours from multiple angles and within the larger enclosure context. View from Nest camera is shown in Figure 2.

### 2.4. Hardware and Software

**Figure 3:**
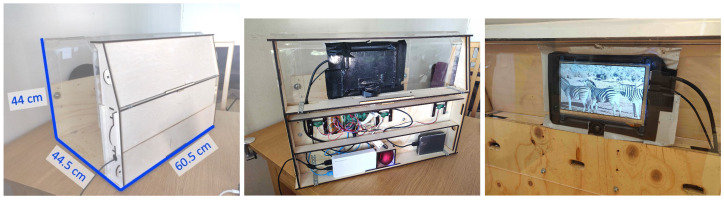
The enrichment device. Left: measurements from the outside, Middle: with the side panel removed to show the technology and screen, Right: view of the screen with the camera below from inside the system.

The enrichment system consisted of the hardware, the software and the visual enrichment stimuli. The device itself and interaction mechanism has been previously used with this troop, shown to have no adverse effects and successfully capture their interactions [7]. The system has a screen screen on one side (7-inch HDMI LCD Rev2.1) as well as a camera (Raspberry Pi Camera V2.1) positioned below the screen (view from camera presented in Figure 2). Three infrared (IR) distance sensors (SHARP GP2Y0A41SKOF 4 -30 cm) are placed within the wall underneath the screen. This system was controlled by a Raspberry Pi 3 (code available in Appendix A). The system was powered through a bite-proofed cable across the top of the enclosure to avoid daily power maintenance (batteries or otherwise). The final system and its components are presented in Figure 3.

There were several possibilities for forming an interaction mechanism for the sakis that have been done with primates before, such as tracking through gaze [43], posture [44], proxemics [7], head-positioning [45], and physical touch screens [19,46,47]. For the device, we used proxemics to reduce any training needed with physical interfaces following prior requirements [41]. Additionally, proxemics has been shown to work with this troop [7] thus is a viable and tested method. For proxemic tracking we used three infrared (IR) distance sensors that created an activation area inside the system. The IR distance detectors were placed at a height where the sakis could trigger the device, but it was unlikely that the other monkeys co-habiting in the enclosure could. We did not want other species of monkeys other than the sakis to use the system to control the research variables.

The software of the system was written in Python running on the Raspberry Pi. This controlled the delivery of visual stimuli clips (MPEG-4), the recording, tracking and processing of the input from the sensors and the saving of the data both locally and online. Due to the bespoke build of the system’s hardware and software the approximate cost of this device is 180 euros at the time of publishing. Further, due to the autonomy and remote access capabilities, the device only required the zoo to install and plug in the system resulting in minimal cost of time for the zoo curators.

### 2.5. Visual Stimuli

**Figure 4:**
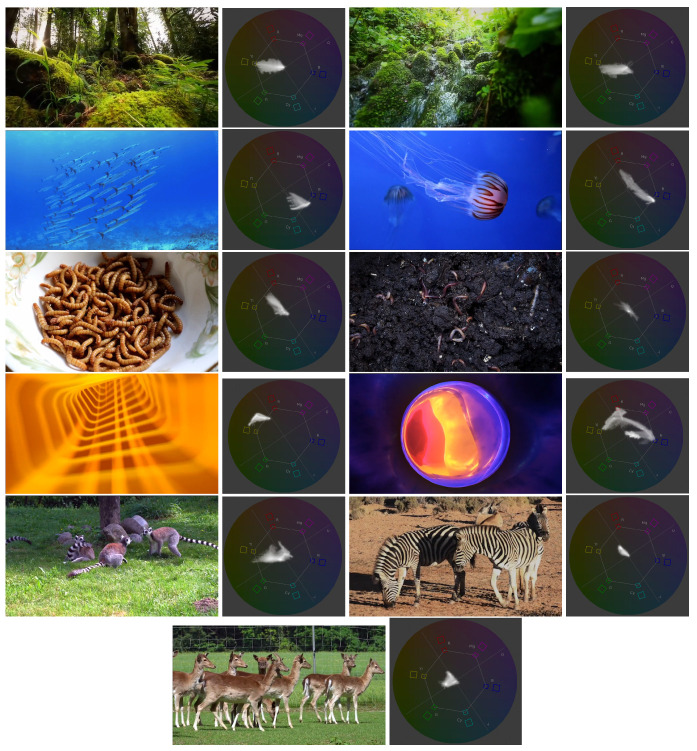
Stills of the videos played to sakis including Adobe Premier Pro vectorscope of hue and saturation. Rows 1: forest video, 2: underwater video, 3: abstract video, 4: worms video, and 5-6: animals video.

As no prior work has been undertaken on sakis’ vision beyond Villani’s [39] work, which found that they are able to differentiate between colours, no reasonable predictions could be made for selecting the visual stimulus to show. Thus, the selected videos were chosen to have varied content (both familiar and unfamiliar to the sakis), different types of movement, and different colour spectra. We used videos of forest, underwater, worms, abstract content and animal videos with no sound. Stills of these videos, and Adobe Premier Pro vectorscope analysis that aided in selecting the colour schemes, can be seen in Figure 4 and links to the full videos are in Appendix B. The contents of videos are summarised in Table 1. Each video was 30 s long, and would loop the same video if the sakis used the system for longer than this time. This length was chosen as the sakis’ previous interactions with digital enrichment have averaged at 4 s [7].

### 2.6. Terminology of Interaction and Activation

Within this paper, an activation results from a saki being identified within the tracking space and is termed as an *interaction*. We also use the term *preference* when the sakis have higher interaction with a certain visual stimuli. However, applying these terms of *interaction* and *preference* to animals need further expanding. Taking the typical Human-Computer Interaction definition of interaction is not applicable to animals [48] as it is unknown to what degree an animal can meaningfully interact, and what their intentions and understanding are with computer enabled enrichment technology [7]. As such, what preferences and the association of liking can be drawn from such an unknown interaction is questionable when it is unknown what goals, interactions, and perceptions the animal user has. While researchers have investigated usability [49], preference [7] and interactions [48,50] with non-human animals with the context of computer devices and interfaces, it is an incomplete science that needs to have leeway for misunderstandings and misconceptions when interpreting a non-human species. In other words, the human idea of computer *interaction* and *preference* is not directly comparable to the non-human animal species with computer-enabled systems. Thus, as Piitulainen and Hirskyj-Douglas note [7], it is possible that the monkey’s interactions with computer devices could be both deliberate and/or accidental. This is especially pertinent in screen contexts where the saki could use the device without looking at or engaging with the screen interface.

The enclosed method mitigates against accidental interactions through having the same stimuli in weekly iterations which enables the making of a reasonable guess at the sakis’ intentions, but there is still room for misunderstandings particularly when interpreting their behaviour. Given these complexities, in this paper we use the terms interaction and preference in the broadest sense; preference is drawn from the interaction timing and frequency where the interaction refers only to the activation’s and behaviours observed.

### 2.7. Data Analysis

Over the study, there were no system failures. The process of the data analysis required three stages: (1) cleaning and verification, (2) coding, and (3) comparison. To begin with, the data was cleaned and verified by pairing together the interaction data with the recordings using the recorded interaction time and by analysing the captured footage which was the combination of the recordings from the cameras inside and outside of the device structure. From this data we removed interactions and recordings that were triggered at night time between 20:00 and 05:00. During this time, while the system remained on, it was dark and we were unable to encode behaviours and verify interactions. In total the system recorded 631 interactions between 05:00 and 20:00 over six weeks. From this data we continued to remove a total of 45 interactions and their recordings (two recordings due to it being too dark, 25 recordings were triggered clearly by another monkey in the enclosure other than the sakis and 18 recordings that were triggered by accident by the system). Of the interactions discarded for it being too dark: one was a short 2 s interaction (at 5:44) and the other discarded interaction was long, lasting for over 9 h when a saki slept inside the system (from 17:59 to 03:29). As a result, we were left with 586 interactions and their recordings triggered by the sakis over six weeks.

In the second phase of data analysis, we encoded the recorded video data into various behaviours to develop an ethogram for this troop and computer device. For this, we used an online spreadsheet software. To form the ethogram, the process began by two researchers, who actively worked and were familiar with the sakis, watching the sakis’ behaviour in person and through video and selecting the initial codes: looking, tactile, passing through, social usage, two monkeys, three monkeys, chasing out, grooming, sleeping and sitting. These same researchers then independently encoded ten randomly selected videos for all the codes they saw. The ethogram was scored using frequency. This resulted in 87 % inter-rater agreement, with the disagreements over the codes ‘social usage’ (3/90), ‘looking’ (3/90), ‘passing through’ (2/90), ‘sitting’ (1/90), ‘two monkeys’ (2/90) and ‘tactile’ (1/90). The disagreements over *social usage* were around the monkeys being social with each other or with the device, the *passing through* over how fast/slow the monkey passes through before it is counted as stopping and *two-monkeys* over if both monkeys need to be in the system at once when they interact. Other behaviours were also noticed such as scratching, stretching and viewing out from the edge of the structure and sitting details such as the sitting location and whether facing the screen while sitting. From this, new codes were added and clear definitions created for each code. The final codes of the ethogram can be seen in Table 2. One researcher then again randomly selected another ten videos and both researchers encoded these separately resulting in an inter-rated agreement of 98% (166/170 agreement). One more code was later added during the first week of the study (viewing window) as it was noticed that the sakis would use the plexiglass window within the device to view out of.

Using the ethogram in Table 2, the sakis’ behaviours were then scored from all the videos captured over the study. This produced a total of 586 behavioural occurrences; 67 over no video enrichment, 89 over forest stimulus, 235 over underwater, 119 over worms, 61 over abstract content and 15 over animals. For our last stage of the data analysis, we then compared these scores and interaction times across the different study conditions. Additionally, to put our findings into a wider context, we interviewed the zoo keeper, who is primarily in charge of the sakis’ enclosure, and research director at Korkeasaari on the results. The comparisons made along with the comments as quotes by zoo staff to speculate on the findings are reported in the results section.

For comparison, we compared sakis’ interactions across conditions using daily number of interactions and use time values (summed duration of all interactions during a given day) of each test week. As the number of monkeys varied during the study they were treated as a group in the comparisons. This variation was due to the zoo keepers removing the monkeys from the exhibit to another space. All sakis that were inside the space, regardless of their usage of the system, were treated as a monkey participating in the study. On average, there were seven sakis for week 1 (control week), seven sakis for week 2 (forest), four sakis for week 3 (underwater), three sakis for week 4 (worms), two sakis for week 5 (abstract), and three sakis for week 6 (animals). Hence, we further divided these daily values with the number of monkeys participating in the study a given day. These values (seven values for each test week) are seen to be independent and not paired by day order, as the study weeks were sometimes interrupted, nor participants, since the data is grouped data as individual sakis could not be identified by the system.

Significance of differences between interactions of different conditions was tested with pairwise independent-samples Wilcoxon rank-sum test (H0: There is no difference in a monkey’s daily use time of the device between conditions), which tested whether condition 1 had larger mean than condition 2. Holm method was used to adjust the p-values of the 30 tests made (6 conditions compared against each others’ values). The results are presented in Table 3, where W represents the number of sub-tests that favoured the condition 1 (out of total 49 sub-tests). Effect size is reported as r-value (r = Z/sqrt(N)).

The effect of video stimuli on different behaviours was tested using the relative values of ethogram scores, the proportion of all interactions that included a given behaviour. Independent Wilcoxon rank-sum test was done between control (N = 7) and visual stimuli conditions (N = 35) for each behaviour using the mean of relative ethogram scores of each test day. The relative scores and the magnitude of change caused by visual stimuli are shown in Table 4. For statistical analysis we used R in R Studio IDE software with external packages of Tidyverse (for data manipulation and visualizations; v1.3.0), rstatix (for Wilcoxon tests; v0.6.0), and moments (D’Agostino-Pearson’s test; v0.14).

## 3. Results

The results are split over our two research questions: the first on what visual enrichment do captive sakis trigger quantified by their interactions, and the second on the behavioural effects of the visual enrichment measured using our ethogram. Through this research method, lasting several weeks and consisting of control and various stimuli conditions, we found how the stimuli influenced the sakis’ interactions with the device and how this in turn affected their behaviour.

### 3.1. Sakis Interactions with the Visual Enrichment System

The average time (activity budget) that individual sakis spent with the enrichment system daily when it was playing stimulus was 560 s per day, with an average of 4.8 interactions.

We compared the sakis’ interactions across different visual stimuli conditions (Table 3) using the values of daily use time per monkey presented as weekly averages in Table 5. The test revealed that the sakis used the device significantly more when visual stimuli was *underwater* (M = 1002, SD = 896) and *worms* (M = 1131, SD = 994) over any other stimuli, including the *no stimuli* condition (M = 168, SD = 186) (Table 3, *p* = 0.041, r = 0.734 for underwater over no stimuli, and *p* = 0.029, r = 0.768 for worms over no stimuli). This effect was the largest with the *worms* stimuli over all other videos. The sakis also used the device significantly less when animal videos (M = 10, SD = 22) were playing than *no stimuli* (Table 3, *p* = 0.041, r = 0.773). As these results indicate, the stimuli triggered by a saki influenced the saki’s usage and aversion of the device. The keepers at the zoo speculated both towards the features of the video and the colours present: ’I don’t know if it is just about the movements or does it have to be like we watch black and white vs colour’.

When looking at the sakis’ daily interaction details (Table 5), correlations can be found between frequency, use time and duration of interactions across stimuli types. The interaction frequency of underwater stimuli was almost double to any other stimuli and it had the longest duration of interactions (6001 s). However, the worms stimuli typically had longer duration of interactions (mean = 202 s) than the underwater stimuli (mean = 112 s). Most of the sakis’ interactions were short interactions, they were highly skewed for all conditions (D’Agostino–Pearson’s test for normality skew = 7.823, z = 24.451, *p* ≈ 0).

**Figure 5:**
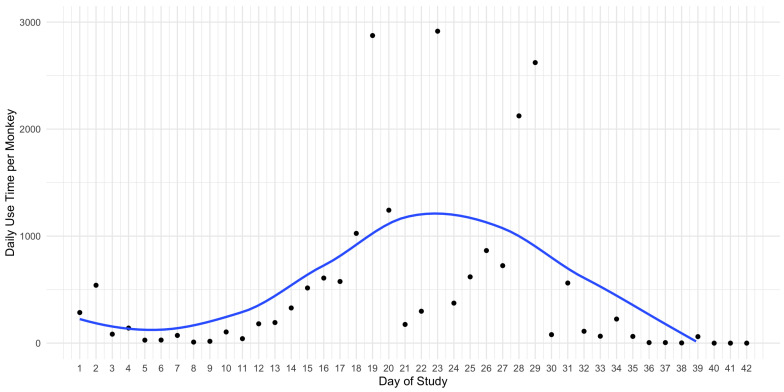
A monkey’s daily use time with the enrichment system with line of best fit.

By visualising the daily usage over the whole study, we found that the sakis’ interaction over time was bell-curved Figure 5. These results could indicate that as the stimuli was prone to the ordering effect, the sakis had inital novelty followed by habituation.

**Figure 6:**
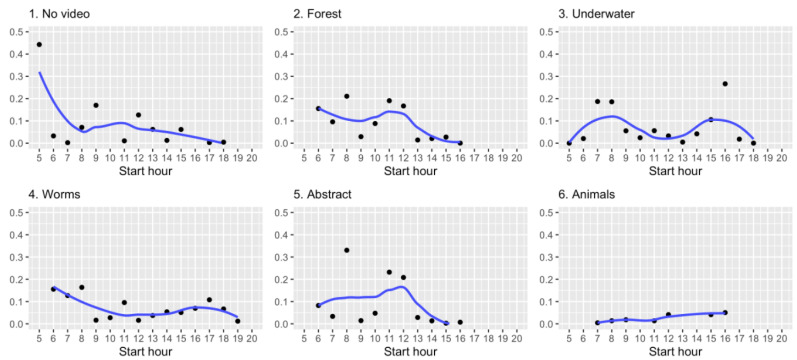
Monkey’s daily usage of the system per hour over different stimuli. Line shows average fitted value.

In addition, we also looked at the sakis’ interactions over the hours of the day. As Figure 6 notes, the sakis typically used the system throughout the day with higher usage in the early morning. This analysis indicates that while the interaction style vary across stimuli conditions, the time of day and the period for how long the device has been used for affect the sakis’ interactions.

### 3.2. Sakis Behaviour with the Visual Enrichment System

To analyse the sakis behaviour across the different conditions we used our ethogram (Table 2) and scored the sakis’ behaviours. The scores are presented as percentages over the total number of interactions of each condition week (Table 6). The keepers at the Korkeasaari Zoo stated about the sakis’ behaviours that ‘there was no noticeable overall behaviour difference of sakis before and after the use of the device’.

As Table 6 demonstrates, while the stimuli conditions influenced various behaviours in the sakis, the only significant result induced by the stimuli conditions was the decrease in scratching (*p* = 0.022, Table 4). Keepers at the Korkeasaari Zoo commented that ‘for primates it’s usually a sign of stress if they scratch a lot’, indicating that scratching can be a displacement behaviour. The stimuli condition also drastically increased the grooming behaviour (152% increase) and social behaviour inside the device (275% increase), although not significantly (grooming *p* = 0.138, social usage *p* = 0.453; Table 4). Nonetheless, as Table 6 shows, most of the behaviours we saw were the monkey passing through the system, although this reduced when the device played visual stimuli (−18.34%; Table 4).

Across the visual stimuli conditions (forest, underwater, worms, abstract and animals) the sakis’ behaviours did change (Table 6). Interestingly, the *looking at screen* behaviour did not increase significantly between the different conditions (*p* = 0.488, 21% control, 17% visual stimuli condition; Table 4) but did vary between the different stimuli (forest 24%, underwater 13%, worms 19%, abstract 23% and animals 7%; Table 6). The keeper who looked after the sakis speculated that ‘If they’re there together, yeah I think it is positive. That they go and sit there together’ and ‘if they groom together, it’s positive. It can also be positive when they groom alone’. These results indicate that visual stimuli might help to induce positive behaviours in sakis.

## 4. Discussion

The findings of this study contribute to visual enrichment for white-faced sakis and to the behavioural effect of controlling a visual enrichment. With increasing usage of visual enrichment systems in zoos for primates, this study advances methods to quantify a monkey’s interactions with computer screens and ways for animals to have control over the computers they use. In the following discussion, we explore these themes situating our findings within computer-enabled visual enrichment for primates.

### 4.1. Sakis’ Interaction with Visual Enrichment Systems

From our system it is evident that the sakis do pay attention, and will trigger screens playing visual content. Additionally, the sakis interactions with the computer-enabled screen device significantly change. However, in this study, it is unclear if it was the video content or the novelty effect of the device that caused this significant effect. Both the content and the novelty effect could lead sakis to seek to trigger the device.

In previous research on animals’ interactions with videos, these two factors (novelty and content) have been shown to influence an animal’s preferences for visual stimuli [33]. In particular, prior work has shown that primates become habituated to videos shown repeatedly [51,52], even if given control over the screens turning on and off [38]. This could also be the case for the sakis, with them becoming habituated to the device, even with the content changing weekly. Further research is needed around creating visual enrichment systems for monkeys that allow for the element of choice and for the sakis to connect (in some sense) the stimulus with the trigger behaviour, without becoming habituated to the devices, process, or stimuli.

Looking at how the sakis interacted with the videos, if the videos motivated the sakis’ behaviour, we do not know what features, colours, references, or other aspects of the content could have caused this. Further research is needed around sakis’ preferences of visual stimuli, such as those noted prior with other monkeys e.g., movement speed [32], colour [26], and sex differences [33]. Additionally, as the sakis’ usage of the device differed in terms of the time of the day, future enrichment systems that use visual stimuli also need to allow for this variability in time periods.

Ultimately, regardless of the trigger motivator, the sakis did use the enrichment device. This study demonstrates a viable method that allow sakis the freedom, opportunity and choice to decide if and when to trigger visual stimuli and a way for quantifying this interaction.

### 4.2. What We Learned about Sakis’ Behaviours with Screen Enrichment Systems

Our method enables effective tracking of the sakis’ behaviours. Overall, the sakis had a diverse set of behaviours with the enrichment system which echoes their normal everyday behaviours in both wild and captive scenarios; social usage, sleeping, scratching, chasing, watching and tactile behaviours. The visual enrichment device affected the behaviours of sakis between the different visual and no-visual content with the sakis *scratching* behaviour being significantly reduced with visual stimuli compared to the control condition. As the system design allowed a form of mediated-consent and choice, the sakis could have avoided the computer enrichment system if it was causing distressing behaviours. This could explain why no negative welfare indicators were logged or reported by the keepers. Nonetheless, this does leave open questions as to what is a positive experience and positive welfare indicators with visual enrichment for sakis. The system’s form, placement, the group’s dynamics, or other factors beyond human measurement could have influenced the monkeys’ behaviours.

Although not significant, we noticed that when the system was playing visual stimuli this also increased social usage (48% increase), especially if two sakis were inside the device at once (275.17% increase in social interaction) (Table 4). A similar finding with visual stimuli has been found prior with an orangutan [23], pointing towards the positive effects of visual enrichment for improving welfare, not only for the primate triggering the visual stimuli but for the overall troop.

It is also worth noting the different ways the sakis engaged in the visual stimuli. When a saki sat in the device form while stimuli were playing, it was not always in front of the screen, but also in the middle or on the edge of the device, facing away from the screen. Further, between the control condition and the stimuli condition the *looking* behaviour of the sakis decreased, even though other behaviours and frequency of interactions with the device changed. Typically, devices developed for primates to view and trigger screens are designed for them to be positioned in front of the screen. However, our system has revealed that how a saki interacts with screens is not always in this human-like mannerism of directly facing the screen. As such, classifying *looking* as direct screen watching with sakis as a form of interaction in itself might not be appropriate.

Instead, we propose that, for monkeys and digital visual interfaces, we need to expand on what we classify as interaction beyond looking, touching, and other human-akin factors and measurements. As this work shows, we need to begin exploring how monkeys wish to interact by allowing them to choose their interactions and freeform their own behaviours and ways of using computer-enabled enrichment devices. While much has been made of the need to expand what interaction means for dogs [48] and engagement for monkeys [7], we note that we also need to open-up what we see as animal-computer interaction and how to quantify this. Through developing new methods, such as the one presented here, we can traverse, learn, and uncover how monkeys use visual stimuli of their own volition. From their usage, and data held within this paper, we can begin to form a foundation to build computer devices informed by the monkeys themselves. In this way, both the technology and the method for quantifying and measuring what we as humans see as animals’ interaction can be made to be animal-centric.

### 4.3. Future Work and Limitations

Whilst our study provides significant steps forward in the form of a wider evaluation of computer-enabled visual stimuli enrichment for primates, there are some limitations to our study. We did not encode all of the sakis’ usage as the enclosure was dark overnight to measure behaviours leaving gaps within our data. In the future, this could be corrected through night vision cameras, or other technology, that would allow 24-h data capture. Although, it is worth noting that in our study the monkeys rarely used the device during this period. Another technological issue we faced is the enclosure camera would not save all the recordings at all times, leaving gaps in the enclosure recordings. For our analysis, at these time frames, we instead only relied upon the recordings from the camera inside the device. We advocate for future technology implementations for zoo-housed animals to have multiple cameras to allow for this perspective and avoid data loss. Looking further at the data analysis, we did not attribute findings to individual monkeys to measure individual differences. This should further be explored in the future as it could be that each monkey has its own personalised way of using the visual device, changing due to the effect of sex, age, family-grouping and other individual differences. This is especially the case for the data where the grouping of the animals is varied, like ours. Further, research could also mitigate against the ordering effect and investigate further into the novelty and habituation effect of computer devices with sakis. We also did not have a follow-up no-stimulus condition to measure the after-effects, as well which would be useful in future studies. Thus the results sit within our study context.

Looking forward, the next stage of research would be to investigate how sakis can choose between visual stimuli to allow them the choice of both activating the device and controlling the content. It would also be pertinent to compare visual stimuli against other forms of enrichment, such as audio, heat, and light, to investigate what enrichment the sakis seek. This would also uncover more of the sakis’ motivation to use the computer-enrichment device. Finally, while the system was only designed and would be triggered by the saki monkey, there were several species of smaller monkeys inside the enclosure. Future work could look at how to build enrichment systems and balance requirements for multi-species usage with varied affordances. Nonetheless, the work described here offers a starting point for further mapping the visual enrichment space for primates.

## 5. Conclusions

This study provides significant insight into how white-faced saki monkeys would trigger different visual stimuli and how a computer device effects the monkeys’ interactions. Our results demonstrate that sakis trigger visual stimuli of underwater and worm significantly more than animal, abstract, forest and the control condition of no stimuli. Additionally, the animal stimuli were triggered significantly less than any other visual stimuli condition. However, due to the curve in the data, this could suggest novelty as a confounding factor. Due to these confounds we are unable to untangle if the stimuli or the novelty factor effected the sakis interaction with the device. Nonetheless, looking at the sakis differences in behaviour with the visual stimuli, the sakis’ *looking* and *facing screen* behaviours did not increase with the visual stimuli vs control conditions. Instead, while the sakis’ behaviour differed across visual enrichment and our control condition, only *scratching* significantly decreased. These findings crystallised the tension of defining animals’ interaction with screens, highlighting the need for a wider definition of interaction and closer scrutiny of novelty and habituation factors for primates with computers. This work builds off prior animal-centred methods that grant animals control over computerised devices and is the first system, that the authors know of, that allows sakis to control their visual enrichment.

## Figures and Tables

**Table 1:** Visual stimuli content.

Video Content	Description		Characteristics
Forest	Two clips filmed in a forest showing scenery, moving slowly.		Slow movement; Green colours; Natural habitat; Calm
Underwater	Two clips filmed underwater. One showing a school of barracudas and the other a jellyfish with bright colour.		Active movement; Shades of blue and bright red colour; Unfamiliar environment; Target to follow
Worms	Two clips: earthworms in soil and a white bowl full of mealworms.		Earthy colour shades; Recognisable objects for the sakis (part of their diet)
Abstract	Two clips: orange-coloured simulation of moving through the structure, and a ball-shaped object with changing bright colours.		Colourful; Movement; Unrecognisable content
Animals	Three 10-s clips of different animals (zebras, lemurs, deer) socially interacting with each other.		Mild colors; Not familiar animals; Social behaviour

**Table 2:** Ethogram of behaviours that the saki monkey had with the device.

Video Content	Description
Passing Through	Monkey passes through the device without stopping at any point or interacting in any form
Viewing out	While inside located at one of the edges of the device, monkey is looking out to other parts of the enclosure
Looking screen	The monkey is seen to look at the screen at some point during triggering
Tactile on wall	The monkey touches anywhere on the wall where the screen resides or the screen as an individual behaviour
Viewing window	The monkey pauses, sits and/or stops to look out of the perspex plastic to the rest of the enclosure
Social Usage	During interaction, multiple monkeys interacting with each other but only one is inside the device at once
Social All Inside	During interaction multiple monkeys inside the device at once
Two Monkeys Usage	Two monkeys inside the device at once
Three Monkeys Usage	Three monkeys inside the device at once
Chasing Out	Chasing another monkey out of the system
Grooming	Grooming together or alone
Sleeping	Seen sleeping by lying down and eyes closed
Scratching	Rubs with its fingers and nails somewhere on itself in a quick manner
Stretching	Stretching
Sitting	Sitting down in the device
Sitting Facing Screen	Sitting down facing the screen within the device
Sitting in the Middle	Sitting location is inside in the middle of the device
Sitting on the Edge	Sitting location is on one of the edges of the device
Sitting on Both	During the interaction, sitting both on middle and edge locations

**Table 3:** Wilcoxon rank-sum test results and effect size for total use time per day per monkey, * <0.05.

Condition 1 (N = 7)	Condition 2 (N = 7)	W	Adj. *p*-Value	Effect Size (r)
No video	Forest	27	1.000	ns
No video	Underwater	3	0.041 *	0.734
No video	Worms	2	0.029 *	0.768
No video	Abstract	19	1.000	ns
No video	Animals	47	0.041 *	0.773
Forest	Underwater	3	0.041 *	0.734
Forest	Worms	1	0.018 *	0.803
Forest	Abstract	16	1.000	ns
Forest	Animals	46	0.049 *	0.738
Underwater	Worms	22	1.000	ns
Underwater	Abstract	40	0.265	ns
Underwater	Animals	49	0.029 *	0.841
Worms	Abstract	41	0.227	ns
Worms	Animals	49	0.029 *	0.841
Abstract	Animals	49	0.029 *	0.841

**Table 4:** Behaviour scores for control and experimental conditions. Percentages are calculated over the total number of interactions for the condition divided by the number of weeks.; the difference is the magnitude of change; * <0.05.

	Control	Visual Stimuli	Difference	*p*-Value
Passing through	59.70%	48.75%	−18.34%	0.397
Viewing out	35.82%	39.50%	10.27%	0.673
Looking screen	20.90%	17.34%	−17.03%	0.488
Tactile on wall	16.42%	7.51%	−54.26%	0.193
Viewing window	16.42%	19.85%	20.89%	0.646
Social usage	5.97%	8.86%	48.41%	0.453
Social all inside	1.49%	5.59%	275.17%	0.253
Two monkeys	7.46%	9.06%	21.45%	0.555
Three monkeys	0.00%	0.39%	NA	0.552
Chasing out	0.00%	0.19%	NA	0.701
Grooming	5.97%	15.03%	151.76%	0.138
Sleeping	4.48%	8.29%	85.04%	0.694
Scratching	28.36%	11.37%	−59.91%	0.022 *
Stretching	8.96%	5.78%	−35.49%	0.898
Sitting	38.81%	45.47%	17.16%	0.879
Sitting facing screen	19.40%	21.97%	13.25%	1.000
Sitting on middle	7.46%	13.68%	83.38%	0.406
Sitting on edge	16.42%	16.38%	−0.24%	0.224
Sitting on both	14.93%	15.41%	3.22%	0.619

**Table 5:** Interaction details per monkey.

		Frequency		Avg Use Time (s)				Duration (s)				
		No. per Day		Weekly	Daily	Daily SD		Mean	Median	Std	Longest	Shortest
No video		1.47		1179	168	186.63		115.22	5	429.23	3281	1
Forest		1.82		874	125	116.48		68.78	3	179.92	1065	1
Underwater		10.02		7016	1002	896.45		112.29	4	520.62	6001	1
Worms		5.61		7919	1131	994.39		202.01	27	432.82	2737	1
Abstract		5.67		3726	523	938.32		93.03	11	217.18	1073	1
Animals		0.71		73	10	22.29		14.53	3	44.17	174	1

**Table 6:** Percentage of sakis behaviour with each visual stimuli condition.

	Control	Forest	Underwater	Worms	Abstract	Animals
	(Total 67)	(Total 89)	(Total (235)	(Total 119)	(Total 61)	(Total 15)
Passing through	59.70%	59.55%	51.49%	36.97%	39.34%	73.33%
Viewing out	35.82%	21.35%	39.15%	52.94%	44.26%	26.67%
Looking screen	20.90%	23.60%	13.19%	19.33%	22.95%	6.67%
Tactile on wall	16.42%	8.99%	5.96%	11.76%	3.28%	6.67%
Viewing window	16.42%	17.98%	16.60%	27.73%	24.59%	0.00%
Social usage	5.97%	16.85%	8.94%	5.88%	4.92%	0.00%
Social all inside	1.49%	8.99%	7.66%	2.52%	0.00%	0.00%
Two monkeys	7.46%	16.85%	9.36%	5.88%	4.92%	0.00%
Three monkeys	0.00%	1.12%	0.43%	0.00%	0.00%	0.00%
Chasing out	0.00%	0.00%	0.00%	0.84%	0.00%	0.00%
Grooming	5.97%	6.74%	9.79%	29.41%	22.95%	0.00%
Sleeping	4.48%	5.62%	5.96%	15.97%	8.20%	0.00%
Scratching	28.36%	15.73%	5.11%	16.81%	21.31%	0.00%
Stretching	8.96%	13.48}	4.26%	3.36%	6.56%	0.00%
Sitting	38.81%	37.08%	43.40%	56.30%	52.46%	13.33%
Sitting facing screen	19.40%	22.47%	17.87%	30.25%	24.59%	6.67%
Sitting on middle	7.46%	13.48%	11.06%	17.65%	19.67%	0.00%
Sitting on edge	16.42%	11.24%	17.45%	20.17%	14.75%	6.67%
Sitting on both	14.93%	12.36%	14.89%	18.49%	18.03%	6.67%

## Data Availability

The data and code are available in Appendix A.

## Appendix A

Data and code are available on Github: https://version.aalto.fi/gitlab/kankaav4/sakis-video-tunnel.

## Appendix B

**Table A1:** Sources of video footage shown to sakis.

Video	Details	Link to Source
Forest clip 1	At 0:12–0:27	https://www.youtube.com/watch?v=Kb8CW3axqRE
Forest clip 2	At 13:02–13:17	https://www.youtube.com/watch?v=Kb8CW3axqRE
Underwater clip 1	Cut of 14 s	https://www.videvo.net/video/school-of-barracuda/4072/
Underwater clip 2	Cut of 18 s	https://www.pexels.com/video/a-group-of-jellyfish-swimming-underwater-at-display-in-an-aquarium-3297378/
Worms clip 1	Cut of 18 s	https://www.pexels.com/video/earthworms-burrowing-under-the-compost-soil-3046307/
Worms clip 2	At 1:18–1:21; 1:29–1:39	https://www.youtube.com/watch?v=ux3vd0zIg78
Abstract clip 1	Cut of 10 s	https://pixabay.com/videos/tunnel-wormhole-abstract-art-43781/
Abstract clip 2	Cut of 20 s	https://pixabay.com/videos/tunnel-yellow-abstract-background-12904/
Animals clip 1	Cut of 10 s	https://pixabay.com/videos/roe-deer-flock-hirsch-wildlife-park-39981/
Animals clip 2	Cut of 10 s	https://pixabay.com/videos/lemur-ape-madagascar-primates-13991/
Animals clip 3	Cut of 10 s	https://www.pexels.com/video/a-group-of-zebras-in-an-open-field-3775802/
